# Cardiac Fibrosis Alleviated by Exercise Training Is AMPK-Dependent

**DOI:** 10.1371/journal.pone.0129971

**Published:** 2015-06-12

**Authors:** Xiaowei Ma, Yongnan Fu, Han Xiao, Yao Song, Ruifei Chen, Jing Shen, Xiangbo An, Qiang Shen, Zijian Li, Youyi Zhang

**Affiliations:** 1 Institute of Vascular Medicine, Peking University Third Hospital, Key Laboratory of Cardiovascular Molecular Biology and Regulatory Peptides, Ministry of Health, Key Laboratory of Molecular Cardiovascular Sciences, Ministry of Education and Beijing Key Laboratory of Cardiovascular Receptors Research, Beijing, China; 2 The First Affiliated Hospital of Nanchang University, Nanchang, China; Rutgers New Jersey Medical School, UNITED STATES

## Abstract

Regular exercise can protect the heart against external stimuli, but the mechanism is not well understood. We determined the role of adenosine monophosphate-activated protein kinase (AMPK) in regulating swimming exercise-mediated cardiac protection against β-adrenergic receptor overstimulation with isoproterenol (ISO) in mice. Ten-week-old AMPKα2^+/+^ and AMPKα2-knockout (AMPKα2^-/-^) littermates were subjected to 4 weeks of swimming training (50 min daily, 6 days a week) or housed under sedentary conditions. The mice received daily subcutaneous injection of ISO (5 mg/kg/d), a nonselective β-adrenergic receptor agonist, during the last 2 weeks of swimming training. Swimming training alleviated ISO-induced cardiac fibrosis in AMPKα2^+/+ ^mice but not AMPKα2^-/- ^mice. Swimming training activated cardiac AMPK in AMPKα2^+/+ ^mice. Furthermore, swimming training attenuated ISO-induced production of reactive oxygen species (ROS) and expression of NADPH oxidase and promoted the expression of antioxidant enzymes in AMPKα2^+/+^ mice but not AMPKα2^-/- ^mice. In conclusion, swimming training attenuates ISO-induced cardiac fibrosis by inhibiting the NADPH oxidase–ROS pathway mediated by AMPK activation. Our findings provide a new mechanism for the cardioprotective effects of exercise.

## Introduction

Cardiac fibrosis is a leading cause of the etiology of heart diseases. Among pathophysiological factors leading to cardiac fibrosis, the activation of the sympathetic nervous system and the consequent release of catecholamines, which stimulate the β-adrenergic receptors in the heart, are very important. Isoproterenol (ISO), a nonselective β-adrenergic receptor agonist, can robustly induce cardiac fibrosis in animal models. As well, transgenic mice with cardiac overexpressing β-adrenergic receptors showed cardiac fibrosis [1]. Exercise training has been shown to reduce fibrosis and matrix metalloproteinase dysregulation in the heart of aged rats [2]. However, the mechanism by which exercise training alleviates cardiac remodeling remains elusive.

Adenosine monophosphate-activated protein kinase (AMPK) is an evolutionarily conserved serine/threonine protein kinase that is a master regulator of energy status from the single-cell to whole-body levels [3]. AMPK can be activated by conditions that increase intracellular AMP such as exercise [4], metformin [5], and hypoxia [6]. AMPK activation instigates a series of signaling events which ultimately regulate the glucose, cholesterol, and fatty acid metabolism [7, 8]. Recent studies showed that AMPK is an endogenous protective factor of the heart [9] and that pharmacological activation of AMPK by metformin or AICAR (5-aminoimidazole 1 carboxamide ribonucleoside) could prevent cardiac remodeling and dysfunction [10, 11]. AMPK activation has also been shown in the exercise trained heart [12, 13]. These studies imply that AMPK could play a cardio-protective role in the exercise training.

Reactive oxygen species (ROS) play important roles in the ISO-induced cardiac fibrosis [14, 15]. Increased cardiac hypertrophy and superoxide production were found in the ISO-infused rats [14], which may be due, in part, to the decreased expression of CuZn-superoxide dismutase (SOD) [16]. Indeed, transgenic mice overexpressing β2-adrenergic receptors showed increased ROS production and progressive ventricular dysfunction [15]. In contrast, activation of AMPK can protect the cardiovascular system against oxidative stress through several mechanisms. These include forkhead transcription factor 3 (FOXO3)-induced thioredoxin (Trx) [17], decreased expression of NADPH oxidase (NOX) and 26S proteasome activity [18], and increased expression of peroxisome proliferator–activated response-γ coactivator-1α (PGC-1α) and manganese superoxide dismutase (MnSOD) [19].

In the present study, we aim at identifying the beneficial role of swimming training-activated AMPK in ISO-induced cardiac fibrosis in mice. Our results show that such training attenuated the ISO-induced cardiac fibrosis through an inhibition of NOX and the resulting ROS production by AMPK activation.

## Materials and Methods

### Animal model and drug treatment

The investigation conformed to the Guide for the Care and Use of Laboratory Animals published by the US National Institutes of Health (NIH Publication No. 85–23, revised 1996). Animal experiments were approved by the Committee of Peking University on Ethics of Animal Experiments (LA 2010–048) and were conducted in accordance with the Guidelines for Animal Experiments, Peking University Health Science Center. All efforts have been made to minimize the suffering of mice. Homozygous AMPKα2-knockout (AMPKα2^-/-^) mice in the C57BL/6 background were kindly provided by Dr. Benoit Viollet (Institute National de la Santé et de la Recherche Médicale U567, Paris) and bred in a specific pathogen-free environment under a 12 h/12 h light-dark cycle and received standard rodent food. Male AMPKα2^-/-^ mice and their AMPKα2^+/+^ littermates (10-week old) were bred and randomly assigned to sedentary or swimming groups (n = 24~30 mice in each group). The swimming group was allowed to swim in tanks with 37 cm in diameter and filled water to a depth of ~30 cm [20]. Water temperature was maintained at 34–35°C. The swimming protocol began with 10 min/day and increased by an increment of 10 min/day until the mice swam continuously for 50 min/day, 6 days/week and the total duration was 4 weeks. Both sedentary and swimming groups were randomized to receive ISO (5 mg/kg/day) or vehicle (saline) daily via subcutaneous injection during the last 2 weeks of swimming training.

### Echocardiography and evaluation of left ventricular hemodynamics

Mice were anaesthetized with 1.5% isoflurane (Baxter Healthcare Corporation). Echocardiographic images were obtained with a VisualSonics high-resolution Vevo 770 system (VisualSonics). Two-dimensional parasternal long axis views and short axis views were obtained at the papillary muscle level. Diastolic left ventricular posterior wall thickness (LVPWd) and systolic left ventricular posterior wall thickness (LVPWs) were measured and ejection fraction (EF) and fractional shortening (FS) were then calculated. All measurements were averaged for three consecutive cardiac cycles. To measure aortic and left ventricular (LV) pressure, a 1.4-F micromanometer conductance catheter (SPR-835; Millar Instruments) was introduced through the right common carotid artery into the ascending aorta and then advanced into the left ventricle as described previously [21].

### Histology

Following anaesthesia with intraperitoneal sodium pentobarbital (100 mg/kg), the mice were sacrificed after pedal pinch reflex were completely inhibited. Mouse hearts were then harvested and perfused in retrograde with cold phosphate-buffered saline (PBS), fixed with 4% paraformaldehyde, and embedded in paraffin. Serial sections (6 μM thick) were stained with hematoxylin and eosin (HE) for morphological analysis or Sirius red for detection of interstitial fibrosis. For morphometrical analysis, photographs of left ventricle sections cut from the same location of each heart were observed by use of a Leica Q550 IW imaging workstation. Cardiac collagen volume fraction was calculated as the ratio of stained fibrotic area to that of total myocardial area.

### Quantitative real-time PCR and Western blot analysis

Total RNA was isolated from the heart tissue by the use of Trizol reagent (Invitrogen). Relative quantification by real-time PCR involving SYBR Green was performed by ABI PRISM 7700 Sequence Detection System (Applied Biosystems). The oligonucleotide primer sequences were showed in S1 Table. GAPDH RNA was amplified as an internal control. The extract proteins were separated by 8–10% SDS-PAGE, then transferred to nitrocellulose membranes. The membranes were probed with the antibodies including anti-phospho-AMPK (Thr 172), anti-AMPK (Cell Signaling Technology), anti-NOX4 (Abcam), anti-NOX2, anti-SOD1, anti-SOD2, anti-CAT, anti-eukaryotic translation initiation factor 5 (anti-eIF5) or anti-GAPDH (Santa Cruz Biotechnology).

### Measurement of ROS and malondialdehyde

ROS production was visualized by staining heart tissues with dihydroethidium (DHE) (Invitrogen Molecular Probes). Serial sections (6 μM) of paraformaldehyde-fixed heart tissue were deparaffinized, rehydrated and incubated with 5 μM DHE for 30 min at 37°C under 5% CO_2_. Sections were examined by use of a Leica digital camera mounted on a fluorescent microscope and data were analyzed by use of Leica software. The content of malondialdehyde (MDA) in myocardial tissue was assayed by the use of a commercial kit from Nanjing Jian-cheng Bioengineering Institute.

### Statistical analysis

All data are presented as mean ± SEM. Comparison of groups involved Student’s paired two-tailed *t* test or two-way ANOVA with the Bonferroni test for post-hoc analysis (Prism 4, GraphPad Software, La Jolla, CA, USA). *P*<0.05 was considered statistically significant.

## Results

### Swimming training attenuates ISO-induced cardiac fibrosis

**Fig 1:**
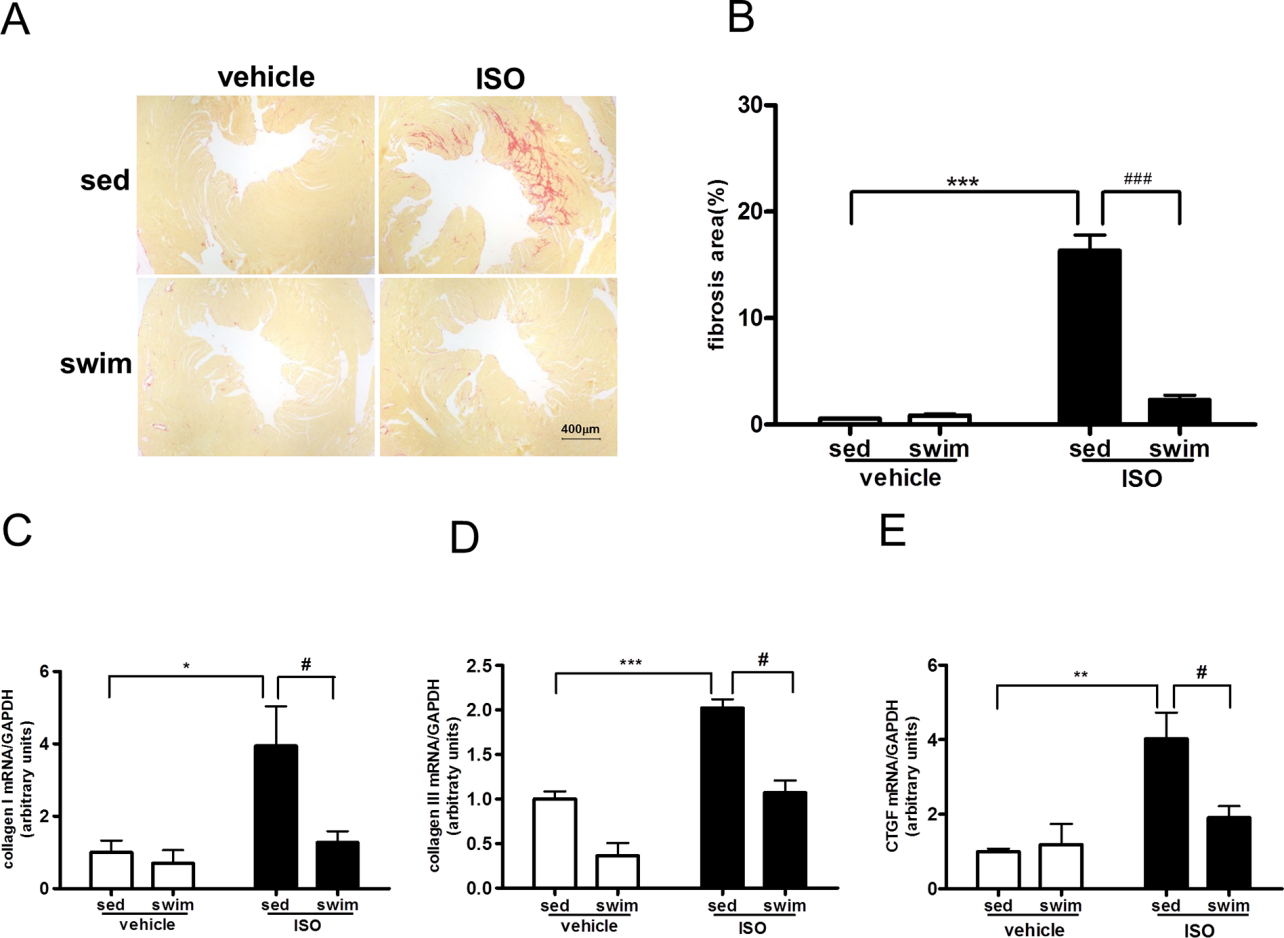
Swimming training attenuated isoproterenol (ISO)-induced cardiac fibrosis in AMPKα2^+/+^ mice. (A) Representative micrographs of Sirius red-stained sections of the left ventricle (LV) (bar = 400 μm). (B) Quantification of mean cardiac interstitial collagen content from Sirius red-stained sections (n = 8~13). RT-PCR analysis of mRNA expression of collagen I (C), collagen III (D), and connective tissue growth factor (CTGF) (E) normalized to that of GAPDH (all n = 4). ^*^ *P*<0.05, ^**^ *P*<0.01, ^***^ *P*<0.001 sedentary (sed)+ISO vs. sed+vehicle; ^**#**^ *P*<0.05, ^**##**^ *P*<0.01, ^**###**^ *P*<0.001 swim+ISO vs. sed+ISO. Data are mean±SEM.

We initially investigated whether swimming training attenuated the ISO-induced cardiac fibrosis. In control experiments shown in Fig 1A, an increase in the deposition of cardiac collagen was seen in the sedentary wild-type, namely AMPKα2^+/+^ mice administered with ISO. In consistent with these pathophysiological changes, ISO administration significantly increased the levels of collagen I, collagen III, and cardiac connective tissue growth factor (CTGF) mRNAs (Fig 1C, 1D and 1E) in the hearts of sedentary mice, indicative of cardiac fibrosis induced by ISO. Swimming training largely abolished all these ISO-induced events involved in cardiac fibrosis. Compared with those in sedentary animals, swimming training decreased the fibrosis area by 86%, collagen I, collagen III, and CTGF mRNAs by 68, 47, and 52%, respectively (Fig 1B, 1C and 1E). Similarly, swimming training also attenuated ISO-induced cardiac hypertrophy in AMPKα2^+/+^ mice (S1 Fig).

### Swimming training-reduced cardiac fibrosis is AMPK dependent

**Fig 2:**
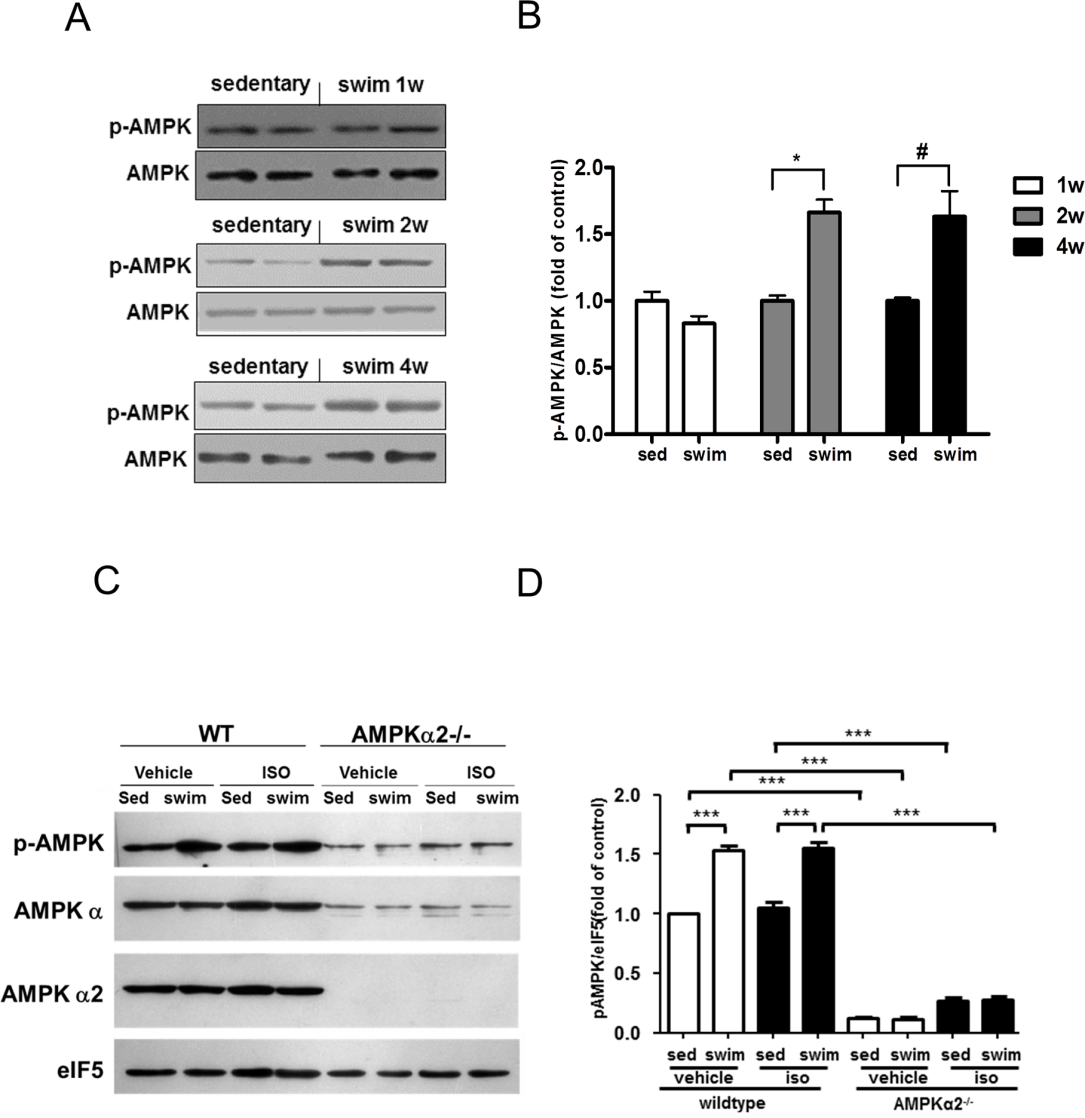
Swimming training activated adenosine monophosphate-activated protein kinase (AMPK) in AMPKα2^+/+^ mouse hearts but not AMPKα2^-/-^ mice. (A) Western blot analysis of protein levels of phosphorylated and total AMPK at different swimming times in AMPKα2^**+/+**^ mice. (B) Quantification of phosphorylated AMPK relative to total AMPK (all n = 4). ^*^ *P*<0.05 swim vs. sed 2 weeks after swimming; ^**#**^ *P*<0.05 swim vs. sed 4 weeks after swimming. (C) Western blot analysis of phosphorylated AMPK, total AMPKα, AMPKα2 and eukaryotic translation initiation factor 5 (eIF5) at the end of swimming training in AMPKα2^**+/+**^ and AMPKα2^**-/-**^ mice. (D) Quantification of phosphorylated AMPK relative to eIF5 (n = 6). ^***^ *P*<0.001. Data are mean±SEM.

To determine whether AMPK is involved in the anti-fibrotic effect exerted by swimming training, we first assessed whether AMPK can be activated in the heart of mice receiving swimming training. Compared to that in animals under sedentary condition, swimming training for 2 and 4 weeks significantly increased the AMPKα phosphorylation at Thr 172, revealing AMPK activation (Fig 2A and 2B). With ISO treatment, swimming training also increased the phosphorylation of AMPK as compared with sedentarism in AMPKα2^+/+^ mice (Fig 2C and 2D). However, swimming training did not increase the phosphorylation of AMPK compared with sedentarism in AMPKα2^-/-^ mice (Fig 2C and 2D). ISO itself had no significantly effect on AMPK activity in AMPKα2^+/+^ mice (Fig 2C and 2D).

**Fig 3:**
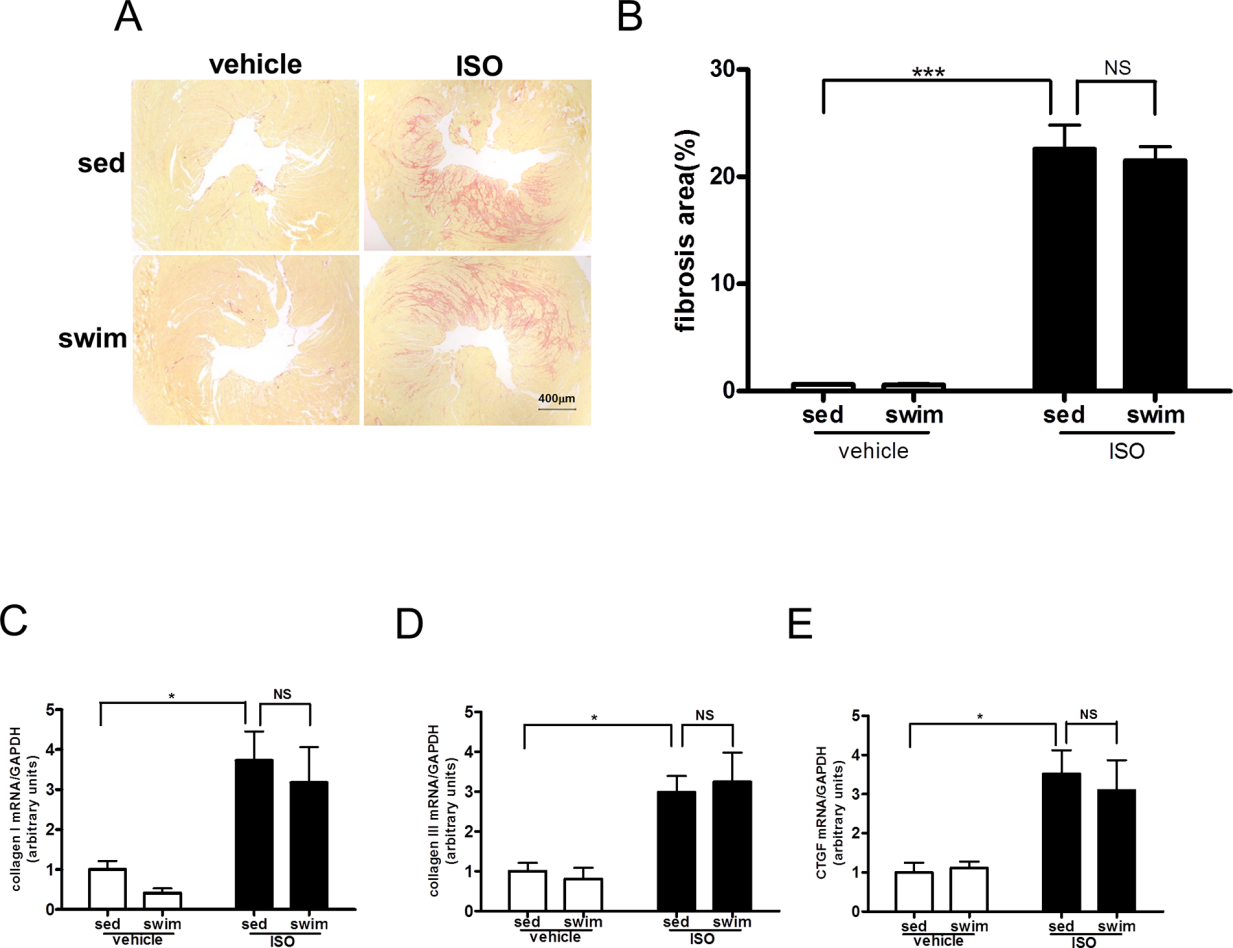
Swimming training cannot attenuate ISO-induced cardiac fibrosis in AMPKα2^-/-^ mice. (A) Representative micrographs of Sirius red-stained sections of the left ventricle (LV) (bar = 400 μm). (B) Quantification of mean cardiac interstitial collagen content from Sirius red-stained sections (n = 8). RT-PCR analysis of mRNA expression of collagen I (C), collagen III (D), and CTGF (E) normalized to that of GAPDH (all n = 5). ^*^ *P*<0.05, ^***^ *P*<0.001 sed+ISO vs. sed+vehicle; NS, not significant. Data are mean±SEM.

We then investigated whether AMPK is critical for the inhibitory effect of swimming training on ISO-induced cardiac fibrosis. With the same protocol as that in Fig 1, AMPKα2^-/-^ littermates were subjected to swimming training or under sedentary in the presence or absence of ISO administration. Among sedentary AMPKα2^-/-^ mice, ISO treatment increased cardiac interstitial fibrosis significantly (22.6%±2.21% vs. 0.6%±0.07%, *P* < 0.001, Fig 3A and 3B), and AMPKα2^-/-^ mice showed more cardiac interstitial fibrosis in response to ISO than AMPKα2^+/+^ mice (22.6%±2.21% vs. 16.3%±1.48%, *P* < 0.01, Figures A and B in S2 Fig). Contrary to the AMPKα2^+/+^ mice (Fig 1A and 1B), swimming training did not improve the ISO-increased interstitial fibrosis in AMPKα2^-/-^ mice (21.5%±1.29% vs. 22.6%±2.21%, *P* > 0.05, Fig 3A and 3B). At the molecular level, ISO treatment increased the content of collagen I, collagen III, and CTGF mRNA in the myocardium of AMPKα2^-/-^ sedentary mice (Fig 3C, 3D and 3E), and the increase of collagen III mRNA was significantly greater in AMPKα2^-/-^ mice than in AMPKα2^+/+^ mice (3.0±0.41 vs. 2.0±0.10-fold of control, *P* < 0.05, Figure D in S2 Fig). In line with the phenotypic changes, swimming training did not reduce the ISO-induced collagen I, III and CTGF mRNAs (Fig 3C, 3D and 3E). Thus, the beneficial effect of swimming training on cardiac fibrosis depends on AMPK. To determine whether gain of function of AMPK could also prevent ISO-induced cardiac fibrosis, we used AICAR, a specific AMPK activator, and we found that AICAR could inhibit ISO induced cardiac fibrosis in AMPKα2^+/+^ mice (Figures A and B in S3 Fig). And in the isolated adult mouse cardiac fibroblasts, AICAR inhibited ISO induced ^3^H-proline incorporation, which suggested that AICAR inhibited ISO induced collagen synthesis in the cardiac fibroblasts (Figure C in S3 Fig). Thus, gain of function of AMPK could prevent ISO-induced cardiac fibrosis *in vivo* and *in vitro*.

We further characterize cardiac functions of AMPKα2^+/+^ and AMPKα2^-/-^ mice treated with or without ISO and receiving with or without swimming training. Functional data showed that left ventricular internal diameter, fractional shortening (FS), left ventricular end-diastolic pressure (LVEDP), the rise and decline of the first derivative of pressure (+d*p*/d*t* and–d*p*/d*t*) were similar in each group of AMPKα2^+/+^ mice (*Table 1*), which indicated that ISO induced cardiac fibrosis without systolic and diastolic dysfunction in AMPKα2^+/+^ mice.

**Table 1:** Cardiac functional data for AMPKα2^+/+^ and AMPKα2^-/-^ mice.

	AMPKα2+/+				AMPKα2-knockout			
	vehicle		ISO		vehicle		ISO	
	sedentary	swim	sedentary	swim	sedentary	swim	sedentary	swim
Echocardiographic data								
*n*	11	11	10	13	12	12	12	13
LVIDd (mm)	3.90±0.07	3.78±0.07	4.07±0.07	4.07±0.05	3.84±0.06	3.81±0.04	4.31±0.10^$$$^	4.06±0.05
LVIDs (mm)	2.82±0.07	2.69±0.07	2.93±0.04	2.97±0.04	2.74±0.05	2.69±0.05	3.41±0.12^$$$^	3.14±0.05
LVAWd (mm)	0.67±0.01	0.66±0.01	0.76±0.01^***^	0.68±0.01^###^	0.69±0.01	0.68±0.01	0.79±0.01^$$$^	0.76±0.01
LVPWd (mm)	0.66±0.01	0.65±0.01	0.76±0.01^***^	0.67±0.01^###^	0.67±0.01	0.67±0.01	0.77±0.01^$$$^	0.73±0.01^@^
FS%	27.7±0.7	28.8±0.7	28.0±0.8	27.2±0.5	28.8±0.6	29.5±0.7	21.0±1.3^$$$^	22.7±0.5
Haemodynamic data								
*n*	13	10	12	12	8	12	12	12
HR (bpm)	469±18	451±13	440±11	420±9	461±18	453±12	461±13	411±3
LVEDP (mmHg)	1.7±0.3	2.2±0.3	2.3±0.3	2.1±0.2	2.7±0.4	2.2±0.2	8.8±0.8^$$$^	8.7±1.0
+d*p*/d*t* (mmHg/s)	6992±237	6700±174	6547±293	6180±142	6947±671	6198±299	4905±273^$$^	4915±268
-d*p*/d*t* (mmHg/s)	-6701±246	-6210±249	-5956±297	-5767±165	-7203±535	-6335±246	-4798±343^$$$^	-4770±278

Values are means±SEM; n, numbers of mice; HR, heart rate; LVIDd, left ventricular end-diastolic inner-dimension; LVIDs, left ventricular end-systolic inner-dimension; LVPWd, diastolic left ventricular posterior wall thickness; LVAWd, diastolic left ventricular anterior wall thickness; FS, Fraction shortening; LVEDP, LV end-diastolic pressure; +d*p*/d*t* and–d*p*/d*t*, the rise and decline of the first derivative of pressure, respectively.

***P<0.001 sed+ISO vs. sed+vehicle in AMPKα2+/+ mice

^###^ *P*<0.001 swim+ISO vs. sed+ISO in AMPKα2+/+ mice

^$$^ *P*<0.01

^$$$^ *P*<0.001 sed+ISO vs. sed+vehicle in AMPKα2-knockout mice

^@^ *P*<0.01 swim+ISO vs. sed+ISO in AMPKα2-knockout mice.

Echocardiography data showed that left ventricular internal diameter increased greatly with ISO than saline treatment, but was not significantly inhibited with swimming training in AMPKα2^-/-^ mice. As well, FS decreased significantly with ISO than saline treatment, but was not prevented with swimming training in AMPKα2^-/-^ mice (*Table 1*). Hemodynamic data showed that LVEDP increased significantly with ISO than saline treatment, but was not inhibited with swimming training in AMPKα2^-/-^ mice. +d*p*/d*t* and–d*p*/d*t* decreased with ISO than saline treatment, but was not prevented with swimming training in AMPKα2^-/-^ mice (*Table 1*). These results indicated that ISO induced cardiac fibrosis with systolic and diastolic dysfunction, and swimming training did not prevent ISO-induced cardiac dysfunction in AMPKα2^-/-^ mice. The ratio of heart weight to tibia length and histology results also suggested that swimming training could not attenuate ISO-induced cardiac hypertrophy in AMPKα2^-/-^ mice (S4 Fig).

### Swimming training attenuated ISO-induced ROS production is AMPK dependent

**Fig 4:**
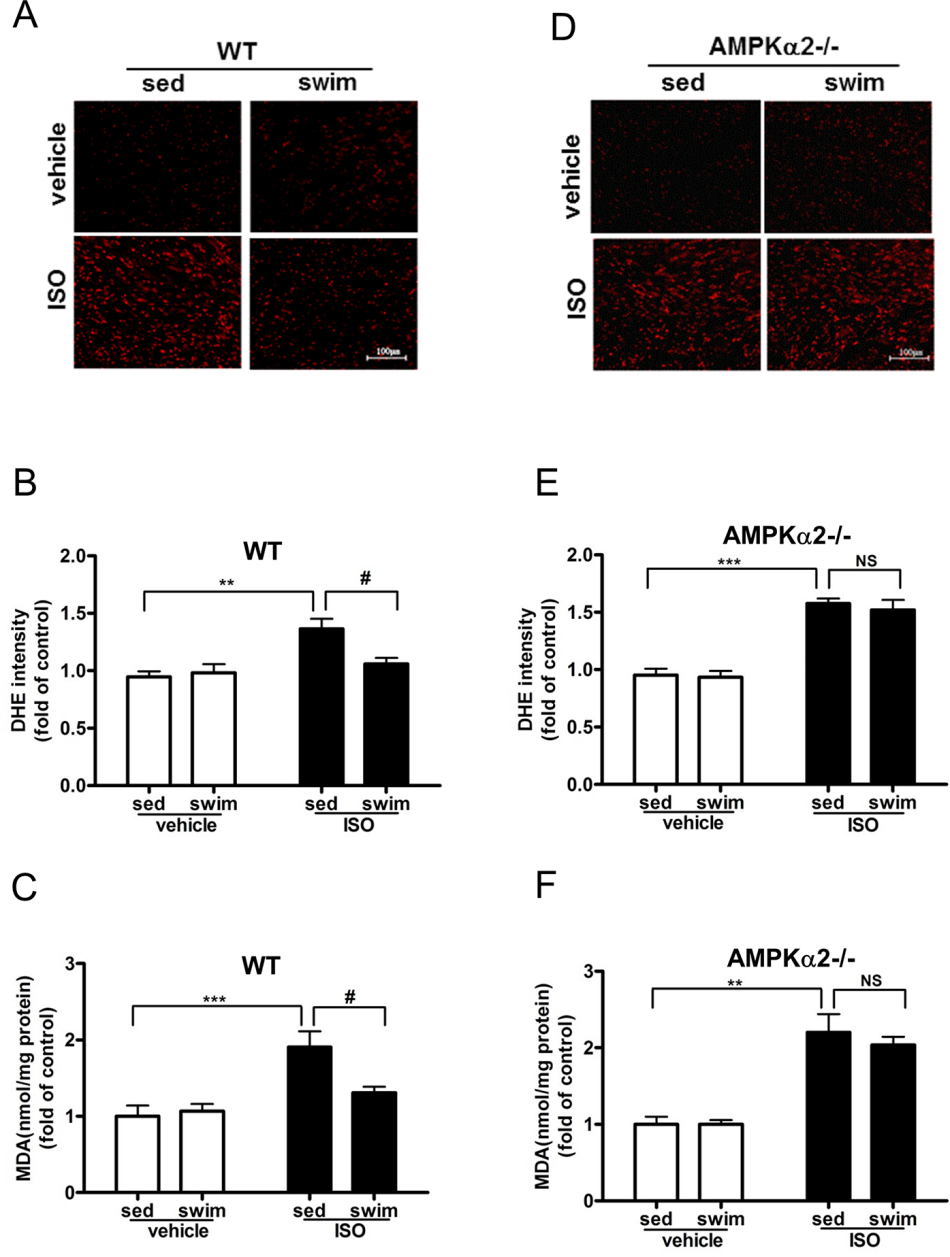
Swimming training attenuated ISO-induced reactive oxygen species (ROS) production in AMPKα2^+/+^ but not AMPKα2^-/-^ mice. (A) Fluorescent microscopy of representative DHE staining for ROS level in LV sections for AMPKα2^**+/+**^ mice (bar = 100 μm). (B) Quantification of fluorescence intensity of DHE in AMPKα2^**+/+**^ sections (n = 6). (C) Malonaldehyde (MDA) content in myocardial tissue in AMPKα2^**+/+**^ mice (n = 6). ^**^ *P*<0.01, ^***^ *P*<0.001 sed+ISO vs. sed+vehicle; ^**#**^ *P*<0.05 swim+ISO vs. sed+ISO. (D) Fluorescent microscopy of representative DHE staining for ROS level in LV sections for AMPKα2^**-/-**^ mice (bar = 100 μm). (E) Quantification of fluorescence intensity of DHE in AMPKα2^**-/-**^ sections (n = 6). (F) MDA content in myocardial tissue in AMPKα2^**-/-**^ mice (n = 6). ^**^ *P*<0.01, ^***^ *P*<0.001 sed+ISO vs. sed+vehicle; NS, not significant. Data are mean±SEM.

Because oxidative stress is crucial in the ISO-induced cardiac fibrosis, we detected ROS formation and the level of MDA, an end product of lipid peroxidation, in the myocardium of AMPKα2^+/+^ and AMPKα2^-/-^ mice subjected to swimming training. ROS production, evidenced by DHE fluorescence intensity, was significantly increased in myocardium of sedentary AMPKα2^+/+^ and AMPKα2^-/-^ mice receiving ISO (Fig 4A, 4B, 4D and 4E). Although AMPKα2^-/-^ mice exhibited more ROS production, there is no significant difference between AMPKα2^-/-^ mice and AMPKα2^+/+^ mice under ISO treatment (1.58±0.04 vs. 1.37±0.09-fold of control, *P >* 0.05, S5 Fig). Swimming training reduced the ISO-increased redox state in the myocardium of AMPKα2^+/+^ (fold of control, 1.06±0.05 vs 1.37±0.09, *P* < 0.05, Fig 4B), but not AMPKα2^-/-^ mice (fold of control, 1.52±0.09 vs. 1.58±0.04, *P* > 0.05, Fig 4E). As well, ISO treatment increased the myocardial MDA level in AMPKα2^+/+^ mice (1.91±0.21 fold of control), which was significantly reduced in animals subjected to swimming training (fold of control, 1.31±0.08 vs. 1.91±0.21, *P* < 0.05, Fig 4C). In contrast, swimming training had little effect on reducing the ISO-increased MDA in AMPKα2^-/-^ mice (fold of control, 2.04±0.11 vs. 2.20±0.24, *P* > 0.05, Fig 4F). Thus, a functional AMPK in the myocardium contributed to the swimming training-reduced the redox state in the mouse heart.

**Fig 5:**
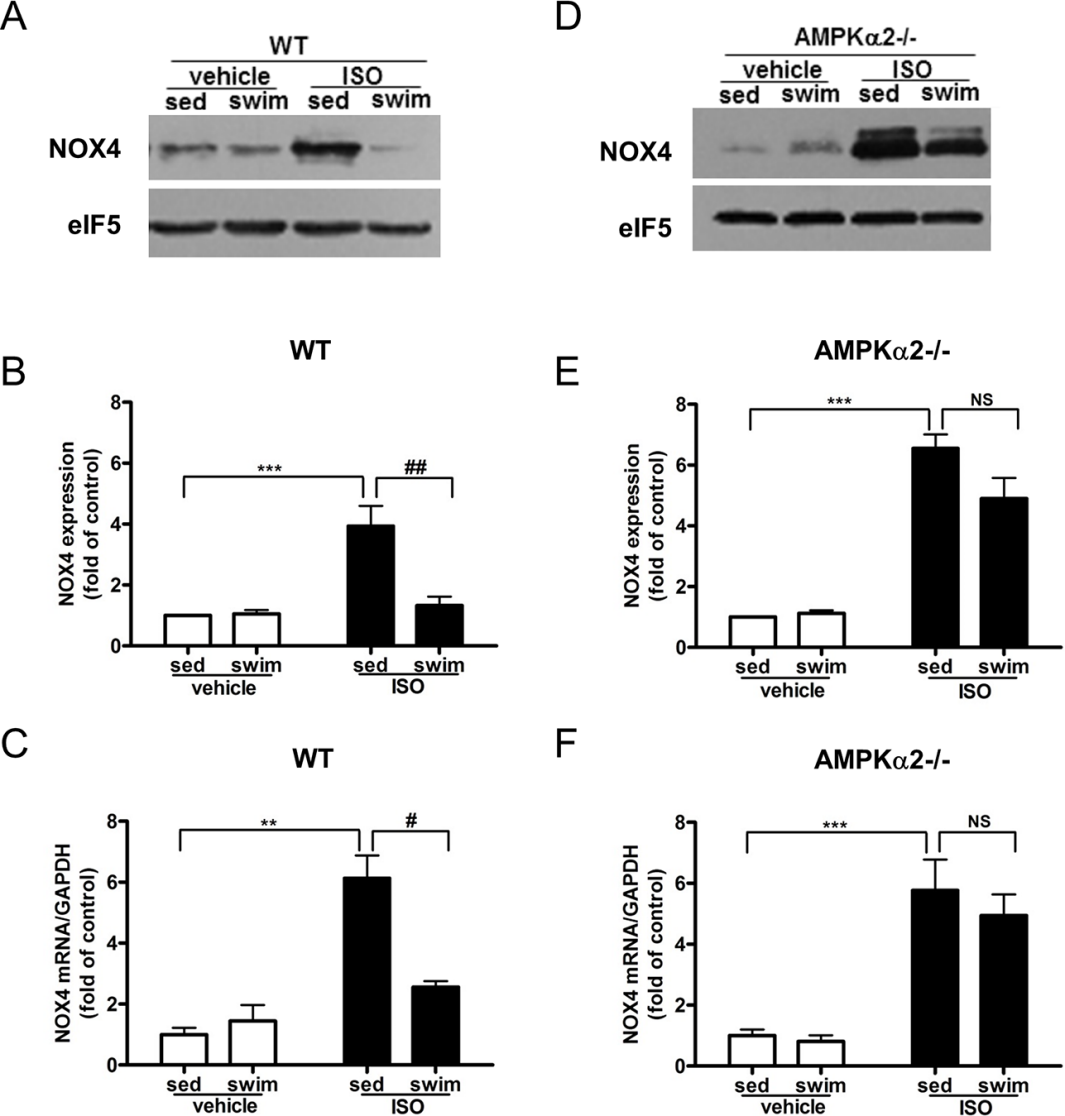
Swimming training attenuated ISO-induced NADPH oxidase expression in AMPKα2^+/+^ mice but not AMPKα2^-/-^ mice. (A) Western blot analysis of myocardial protein levels of NOX4 and eIF5 for AMPKα2^**+/+**^ mice. (B) Quantification of NOX4 relative to eIF5 for AMPKα2^**+/+**^ mice (n = 4). (C) RT-PCR analysis of mRNA expression of NOX4 normalized to that of GAPDH for AMPKα2^**+/+**^ mice (both n = 5). ^**^ *P*<0.01, ^***^ *P*<0.001 sed+ISO vs. sed+vehicle; ^**#**^ *P*<0.05, ^**##**^ *P*<0.01 swim+ISO vs. sed+ISO. (D) Western blot analysis of myocardial protein levels of NOX4 and eIF5 for AMPKα2^**-/-**^ mice. (E) Quantification of NOX4 relative to eIF5 in AMPKα2^**-/-**^ mice (n = 4). (F) RT-PCR analysis of mRNA expression of NOX4 normalized to that of GAPDH for AMPKα2^**-/-**^ mice (n = 6). ^***^ *P*<0.001 sed+ISO vs. sed+vehicle; NS, not significant. Data are mean±SEM.

To clarify the mechanism by which swimming training reduces oxidative stress, we examined the expression of NADPH oxidase, the major enzymatic source of ROS, in the heart of AMPKα2^+/+^ and AMPKα2^-/-^ mice. In particular, the mRNA and protein levels of NOX2 and NOX4, two NADPH oxidase subunits were assessed. Myocardial NOX4 mRNA and protein levels were significantly elevated in ISO-treated AMPKα2^+/+^ mice, which were reversed by swimming training (Fig 5A, 5B and 5C). Although ISO treatment had no effect on myocardial NOX2 protein expression in AMPKα2^+/+^ mice (Figure A in S6 Fig), such treatment markedly increased the expression of NOX2 mRNA, and the increase was significantly reduced by swimming training (Figure B in S6 Fig).

In AMPKα2^-/-^ mice myocardial NOX4 protein and mRNA expression were significantly higher in ISO-administered animals than saline-treated controls (Fig 5D, 5E and 5F). And AMPKα2^-/-^ mice exhibited significantly greater NOX4 protein expression compared with AMPKα2^+/+^ mice (6.56±0.46 vs. 3.94±0.65-fold of control, *P* < 0.01, Figure A in S7 Fig). Contrary to the results seen in AMPKα2^+/+^ mice, swimming training did not significantly attenuate the ISO-induced NOX4 (4.90±0.67-fold vs. 6.56±0.46-fold of control, *P* > 0.05, Fig 5E) and NOX4 mRNA (4.94±0.70-fold vs. 5.77±1.01-fold of control, *P* > 0.05, Fig 5F) in AMPKα2^-/-^ mice. Regarding NOX2, although no significant difference at protein level(Figure C in S6 Fig), ISO treatment significantly increased myocardial NOX2 mRNA expression (Figure D in S6 Fig). Similarly, swimming training did not attenuate the ISO-increased NOX2 mRNA in AMPKα2^-/-^ mice (1.95±0.37-fold vs. 2.21±0.49-fold of control, *P* > 0.05, Figure D in S6 Fig). Thus, the inhibitory effect of swimming training on NADPH oxidase expression was AMPK-dependent.

### Swimming training promoted the myocardial expression of antioxidants

**Fig 6:**
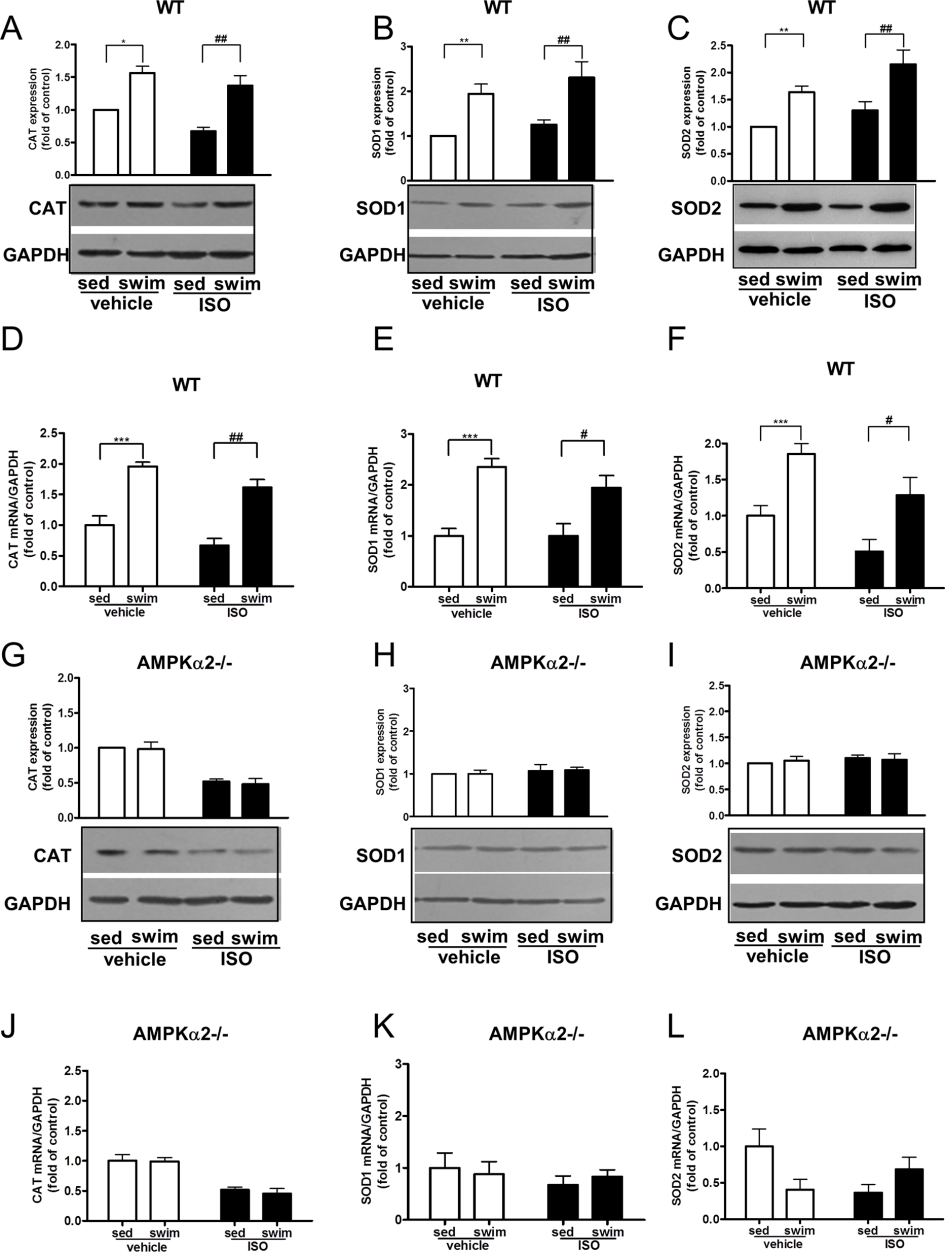
Swimming training promoted the expression of antioxidant enzymes in AMPKα2^+/+^ mice but not AMPKα2^-/-^ mice. Western blot analysis of protein levels of CAT (A), SOD1 (B) and SOD2 (C) in AMPKα2^**+/+**^ mice. RT-PCR analysis of mRNA levels of CAT(D), SOD1(E) and SOD2(F) in AMPKα2^**+/+**^ mice. They are all normalized to level of GAPDH (all n = 5). ^*^ *P*<0.05, ^**^ *P*<0.01, ^***^ *P*<0.001 swim+vehicle vs. sed+vehicle; ^**#**^ *P*<0.05, ^**##**^ *P*<0.01 swim+ISO vs. sed+ISO. Western blot analysis of protein levels of CAT (G, n = 4), SOD1 (H, n = 4) and SOD2 (I, n = 4) in AMPKα2^**-/-**^ mice. RT-PCR analysis of mRNA levels of CAT (J, n = 5),SOD1 (K, n = 7) and SOD2 (L, n = 5) in AMPKα2^**-/-**^ mice. They are all normalized to GAPDH. Data are mean±SEM.

To study whether AMPK is involved in swimming training-induced expression of antioxidant enzymes, we determined myocardial levels of SOD and CAT in AMPKα2^+/+^ and AMPKα2^-/-^ mice. CAT protein expression decreased significantly in AMPKα2^+/+^ mice and AMPKα2^-/-^ mice with ISO treatment, and significantly less in AMPKα2^-/-^ than in AMPKα2^+/+^ mice (Figure A in S8 Fig). CAT mRNA expression decreased significantly in AMPKα2^-/-^ mice with ISO treatment (Figure D in S8 Fig). Although ISO treatment had tendency to decrease CAT mRNA expression, there was no significant difference between ISO and vehicle group in AMPKα2^+/+^ mice (Figure D in S8 Fig). For AMPKα2^+/+^ mice, swimming training significantly increased the myocardial protein and mRNA levels of CAT (Fig 6A and 6D), SOD1 (Fig 6B and 6E), and SOD2 (Fig 6C and 6F) with both saline and ISO treatment. As expected, this increased expression of antioxidants induced by swimming training was much reduced in AMPKα2^-/-^ mice (Fig 6G, 6H, 6I, 6J, 6K and 6L), which suggests that AMPK is involved in swimming training-induced expression of antioxidant enzymes.

## Discussion

Exercise training has been widely studied for protecting ischemic hearts. For animal models with acute myocardial infarction, the beneficial effects of exercise training include, but limit to, reduction in scar size, increase in arteriole density, improved left ventricular remodeling, and increased heart functions [22, 23]. Exercise training also restores a transmural non-uniformity of the myocardium lost during heart failure [24]. Here, we study the molecular basis underlying the beneficial effect of exercise training on ISO-induced fibrosis. Our data show that swimming training alleviates ISO-induced cardiac fibrosis in AMPKα2^+/+^, but not AMPKα2^-/-^ mice, which suggest that this protective effect from swimming training is through an AMPK-dependent mechanism. Metformin, known to activate AMPK, protects heart against ischemia-reperfusion injury in mice [25], inhibits cardiomyocyte apoptosis, and prevents the progression of heart failure in dogs [26]. As well, AMPKα2 deficiency in AMPKα2^-/-^ mice exacerbated pressure overload-induced left ventricular hypertrophy [9]. Previous studies reported that exercise training could activate cardiac AMPK [4, 27], but the possible roles and mechanisms of AMPK in the protective effect of exercise training have yet to be elucidated.

ROS play key roles in ISO/β-adrenergic receptor-mediated cardiac fibrosis [14, 15], and treatment of rats with tempol (an ROS scavenger) prevents cardiac fibrosis, collagen accumulation, and type I collagen mRNA expression induced by ISO infusion [14]. As well, NAC, another ROS scavenger, reduced cardiac fibrosis, suppressed matrix metalloproteinase activity and preserved cardiac function [15]. In the present study, we show that the level of myocardial ROS and MDA increased significantly in all mice after ISO administration. NOX4 is a major source of oxidative stress in the failing heart [28] and increased NOX4 expression enhances mitochondrial oxidative stress and consequent mitochondrial dysfunction, which facilitates electron leakage, thereby increases ROS release [29]. Consistent with our results, myocardial NOX4 mRNA expression was increased in transgenic mice overexpressing β_2_-adrenergic receptor [15]. NF-κB is an important transcription factor to increase NADPH oxidase expression. Wang *et al*. have showed that AMPK activation could reduce NADPH oxidase expression and activity in aortic endothelial cells through inhibiting NF-κB activation [18]. Several studies have showed that AMPK has inhibitory effect on NF-κB activity [30, 31, 32]. Although a single bout of exercise lead to the activation of NF-κB [33], regular exercise training can inhibit NF-κB activity in skeletal muscle, hepatocytes, and endothelial cells. Thus, swimming inhibits NOX4 expression might through AMPK activation and NF-κB inhibition. Although a single bout of exercise increases the ROS level in myocardium, regular exercise is widely considered to enhance antioxidants and suppress superoxide production in the heart tissue [34]. Mechanistically, repeated bouts of aerobic exercise reduced contraction-induced free radical generation and greatly increased total SOD activity and Mn-SOD expression [35]. Similarly, regular physical activity reduces the vascular expression of NADPH oxidase, thus resulting in decreased local ROS generation in patients with coronary artery disease [36].

Transforming growth factor (TGF)-β1 is a potent fibrogenic factor that mediates ECM homeostasis through different mechanisms, for example, by inducing ECM (such as collagens and fibronectin) synthesis [37]. Studies have shown that TGFβ1-Smads signalling pathway are markedly up-regulated at the site of injury after MI [38], in patients suffering from dilated cardiomyopathy [39], and all these conditions are characterised by excessive fibrosis in the heart. Consistently, TGFβ1 overexpression in transgenic mice leads to myocardial fibrosis [40]. ROS are often associated with TGFβ1 signalling. ROS might increase cardiac fibrosis by increasing the production of TGFβ1 [41]. NOX4 has also been shown to mediate TGFβ1-induced conversion of fibroblasts to myofibroblasts by regulating Smad 2/3 activation [42]. Previous studies in our lab showed that the expression of TGFβ1 protein was increased in pressure overload-induced cardiac fibrosis, and metformin inhibited pressure overload-induced TGFβ1 production [43]. In the present study, swimming has tendency to decrease ISO induced TGFβ1 and p-Smad2 in AMPKα2^+/+^ mice. However, swimming has no effect on the ISO induced TGFβ1 and p-Smad2 in AMPKa2^-/-^ mice (data not shown). This suggested that swimming might decrease ISO induced cardiac fibrosis through inhibiting TGFβ1 and p-Smad2 by AMPK activation.

Data acquired in the present study show that swimming training ameliorates ISO-induced fibrosis, NOX4 expression, and ROS production in the myocardium and increases the expression of myocardial antioxidant enzymes, including SOD1, SOD2, and CAT in AMPKα2^+/+^ mice, when compared with AMPKα2^-/-^ littermates. AMPK activity is associated with the redox state in several tissue types in the cardiovascular system. Incubation of human umbilical vein endothelial cells with high level of glucose significantly increased intracellular ROS, which is prevented by aminoimidazole carboxamide ribonucleotide (AICAR) treatment [44]. As well, AICAR activation of AMPK significantly reduces ROS levels caused by palmitic acid in human aortic endothelial cells [17]. In addition, AMPKα2 suppresses NADPH oxidase expression and ROS production in ECs by attenuating the NF-κB-mediated expression of NADPH oxidase [18] as well as inducing manganese SOD and mitochondrial biogenesis via the AMPK-PGC1α pathway [19]. These protective effects of AMPK against ROS in conjunction with our data suggest that AMPK is involved in the protective action of swimming training against ISO-induced ROS formation.

**Fig 7:**
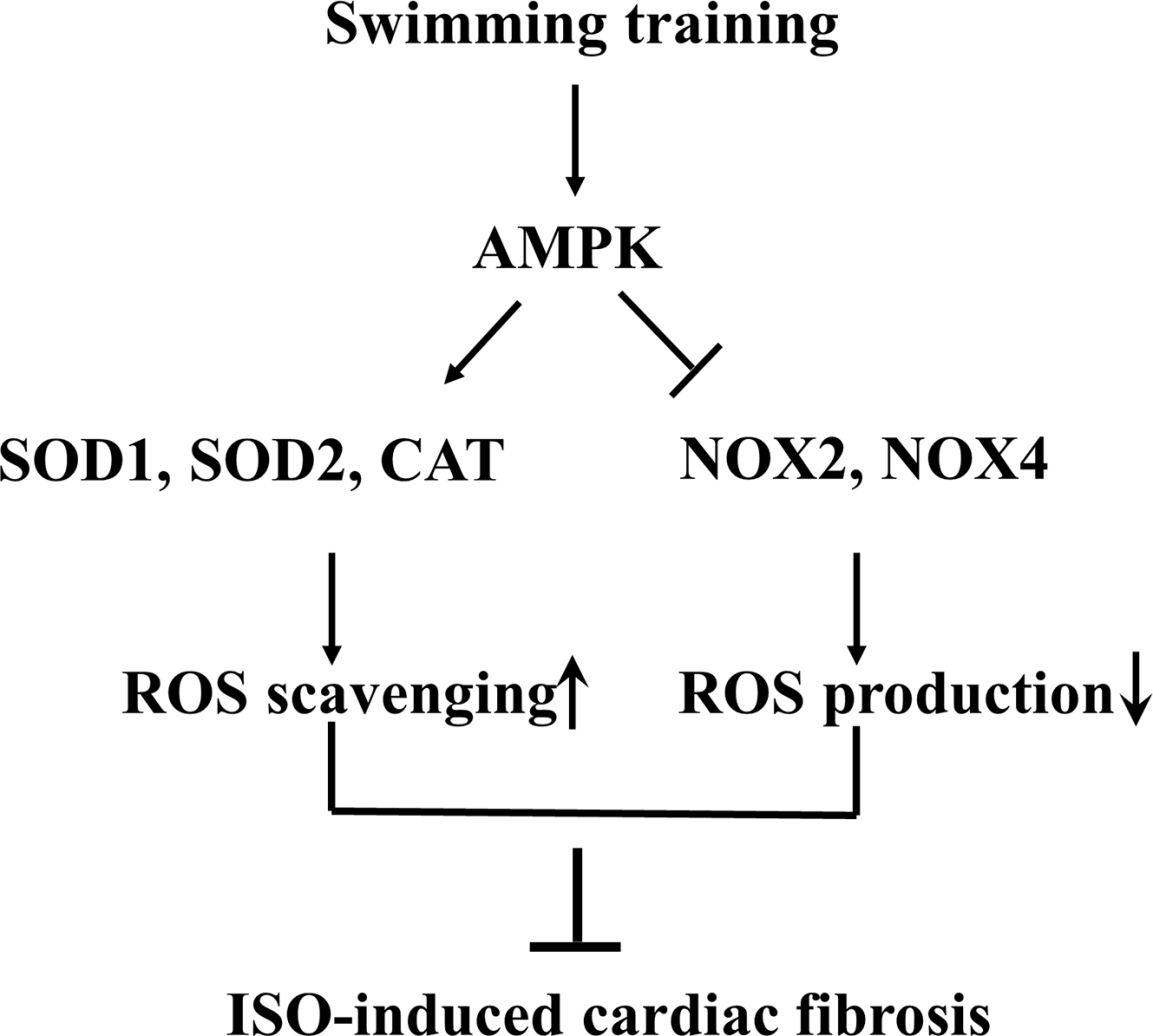
Schematic model of swimming training preventing cardiac fibrosis.

In summary, the present study demonstrated that swimming training inhibited ISO-induced cardiac fibrosis in mice (Fig 7). AMPK-mediated antioxidant defenses (including reduced ROS production and increased ROS scavenging) are a central mechanism for the anti-fibrotic effect of swimming training.

## Supporting Information

S1 Fig Swimming training attenuated isoproterenol (ISO)-induced cardiac hypertrophy in AMPKα2^+/+^ mice. (A) Representative micrographs of echocardiography. (B) The ratio of heart weight (HW) to tibia length (TL) (n = 12). (C) Representative micrographs of HE-stained sections of the left ventricle (LV) (bar = 20 μm). (D) Quantification of mean myocyte cross section area from HE-stained sections (n = 7). ^***^ *P*<0.001 sedentary (sed)+ISO vs. sed+vehicle; ^##^ *P*<0.01, ^###^ *P*<0.001 swim+ISO vs. sed+ISO. Data are mean±SEM.
(TIF)

S2 Fig Isoproterenol (ISO)-induced cardiac fibrosis in AMPKα2^+/+^ mice and AMPKα2^-/-^ mice. (A) Representative micrographs of Sirius red-stained sections of the left ventricle (LV) (bar = 400 μm). (B) Quantification of mean cardiac interstitial collagen content from Sirius red-stained sections (n = 7~13). RT-PCR analysis of mRNA expression of collagen I (C), collagen III (D), and connective tissue growth factor (CTGF) (E) normalized to that of GAPDH (all n = 4). ^*^ *P <* 0.05, ^**^ *P <* 0.01, ISO-treated AMPKα2^-/-^ vs. AMPKα2^+/+^ mice. Data are mean±SEM.
(TIF)

S3 Fig AICAR attenuated isoproterenol (ISO)-induced cardiac fibrosis *in vivo* and *in vitro*. Male AMPKα2^+/+^ mice (10-week old) were pretreated with AICAR (250mg/kg/d) for 3 days and then treated with ISO (5 mg/kg/day) or vehicle (saline) for 7 days. (A) Representative micrographs of Sirius red-stained sections of the left ventricle. (B) Quantification of mean cardiac interstitial collagen content from Sirius red-stained sections (n = 5–8). *P<0.05, ***P<0.001. Data are mean±SEM. (C) AICAR decreased ISO-induced ^3^H-proline incorporation in isolated adult mouse cardiac fibroblasts (CFs). CFs were pretreated with AICAR (10^−4^ mol/L) for 30min and then treated with ISO (10^-5^mol/L). L-[2,3-^3^H] proline were then supplied for 48 hours (n = 4). *P < 0.05 vs. con; ^##^ *P <* 0.01 vs. ISO. Data are mean±SEM.
(TIF)

S4 Fig Swimming training cannot attenuate ISO-induced cardiac hypertrophy in AMPKα2^-/-^ mice. (A) Representative micrographs of echocardiography. (B) The ratio of heart weight (HW) to tibia length (TL) (n = 12). (C) Representative micrographs of HE-stained sections of the left ventricle (LV) (bar = 20 μm). (D) Quantification of mean myocyte cross section area from HE-stained sections (n = 7). ^***^ *P*<0.001 sed+ISO vs. sed+vehicle; NS, not significant. Data are mean±SEM.
(TIF)

S5 Fig Isoproterenol (ISO)-induced reactive oxygen sepcies (ROS) production in AMPKα2^+/+^ mice and AMPKα2^-/-^ mice. (A) Fluorescent microscopy of representative DHE staining for ROS level in LV sections (bar = 100 μm). (B) Quantification of fluorescence intensity of DHE in LV sections (n = 6). (C) Malonaldehyde (MDA) content in myocardial tissue (n = 6). Data are mean±SEM.
(TIF)

S6 Fig Swimming training attenuated ISO-induced NOX2 mRNA expression in AMPKα2^+/+^ mice but not AMPKα2^-/-^ mice. (A) Western blot analysis of myocardial protein levels of NOX2 and eIF5 for AMPKα2^+/+^ mice. (B) RT-PCR analysis of mRNA expression of NOX2 normalized to that of GAPDH for AMPKα2^+/+^ mice (n = 5). ^*^ *P*<0.05, sed+ISO vs. sed+vehicle; ^##^ *P*<0.01, swim+ISO vs. sed+ISO. (C) Western blot analysis of myocardial protein levels of NOX2 and eIF5 for AMPKα2^-/-^ mice. (D) RT-PCR analysis of mRNA expression of NOX2 normalized to that of GAPDH for AMPKα2^-/-^ mice (n = 6). ^*^ *P*<0.05, sed+ISO vs. sed+vehicle; NS, not significant. Data are mean±SEM.
(TIF)

S7 Fig Isoproterenol (ISO)-induced NADPH oxidase expression in AMPKα2^+/+^ mice and AMPKα2^-/-^ mice. Quantification of Western blot for NOX4 (A) and NOX2 (B) relative to eIF5 (both n = 4). RT-PCR analysis of mRNA expression of NOX4 (C) and NOX2 (D) normalized to that of GAPDH (n = 4~6). ^**^ *P <* 0.01 ISO-treated AMPKα2^-/-^ vs. AMPKα2^+/+^ mice. Data are mean±SEM.
(TIF)

S8 Fig Expression of antioxidant enzymes in isoproterenol (ISO)-treated AMPKα2^+/+^ mice and AMPKα2^-/-^ mice. Quantification of Western blot for CAT (A, n = 4), SOD1 (B, n = 5), and SOD2 (C, n = 6) relative to GAPDH. RT-PCR analysis of mRNA expression of CAT (D, n = 5), SOD1 (E, n = 6), and SOD2 (F, n = 5) normalized to that of GAPDH. ^*^ *P <* 0.05, ^***^ *P* < 0.001. Data are mean±SEM.
(TIF)

S1 Table Oligonucleotide primer sequences used for real-time PCR. (DOC)

S1 Methods Supporting information methods. (DOCX)
