# Supplementary figures and images for: Cardiac Fibrosis Alleviated by Exercise Training Is AMPK-Dependent
Source: PLoS One. 2015 Jun 12;10(6):e0129971. doi: 10.1371/journal.pone.0129971 (PMC4466316; doi:10.1371/journal.pone.0129971)

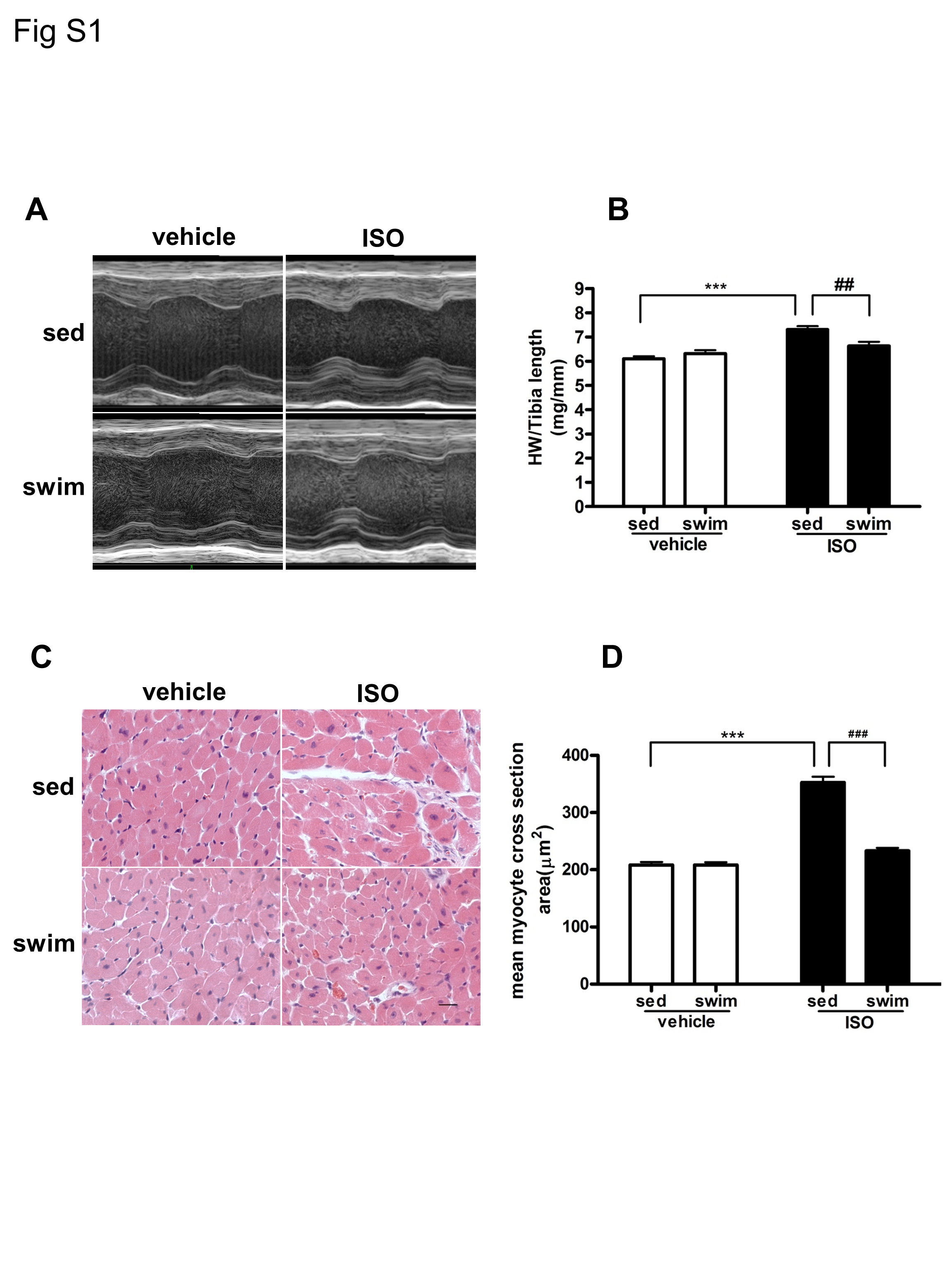

Supplement: S1 Fig — (A) Representative micrographs of echocardiography. (B) The ratio of heart weight (HW) to tibia length (TL) (n = 12). (C) Representative micrographs of HE-stained sections of the left ventricle (LV) (bar = 20 μm). (D) Quantification of mean myocyte cross section area from HE-stained sections (n = 7). *** P<0.001 sedentary (sed)+ISO vs. sed+vehicle; ## P<0.01, ### P<0.001 swim+ISO vs. sed+ISO. Data are mean±SEM. (TIF) [file pone.0129971.s001.tif]

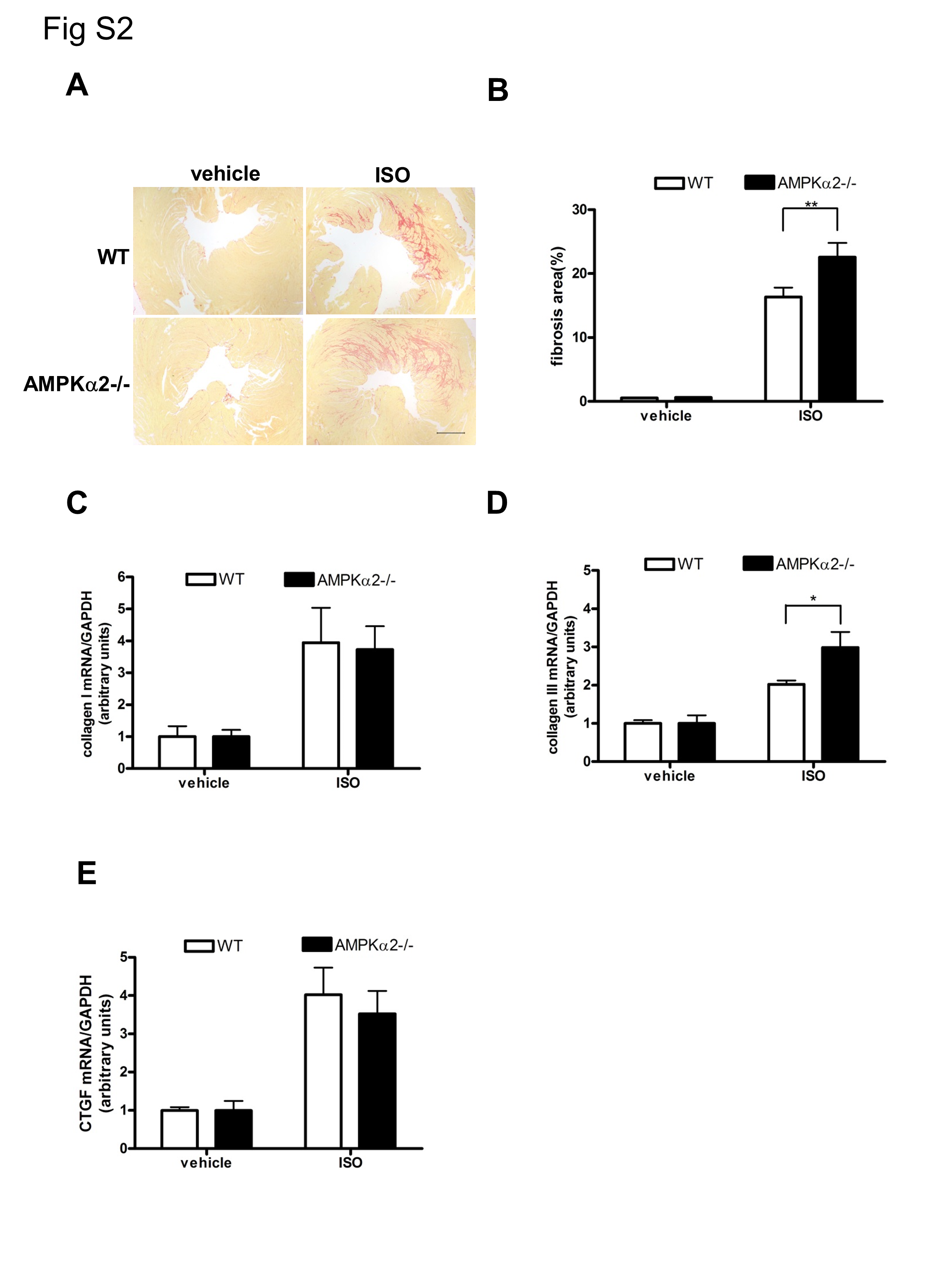

Supplement: S2 Fig — (A) Representative micrographs of Sirius red-stained sections of the left ventricle (LV) (bar = 400 μm). (B) Quantification of mean cardiac interstitial collagen content from Sirius red-stained sections (n = 7~13). RT-PCR analysis of mRNA expression of collagen I (C), collagen III (D), and connective tissue growth factor (CTGF) (E) normalized to that of GAPDH (all n = 4). * P < 0.05, ** P < 0.01, ISO-treated AMPKα2-/- vs. AMPKα2+/+ mice. Data are mean±SEM. (TIF) [file pone.0129971.s002.tif]

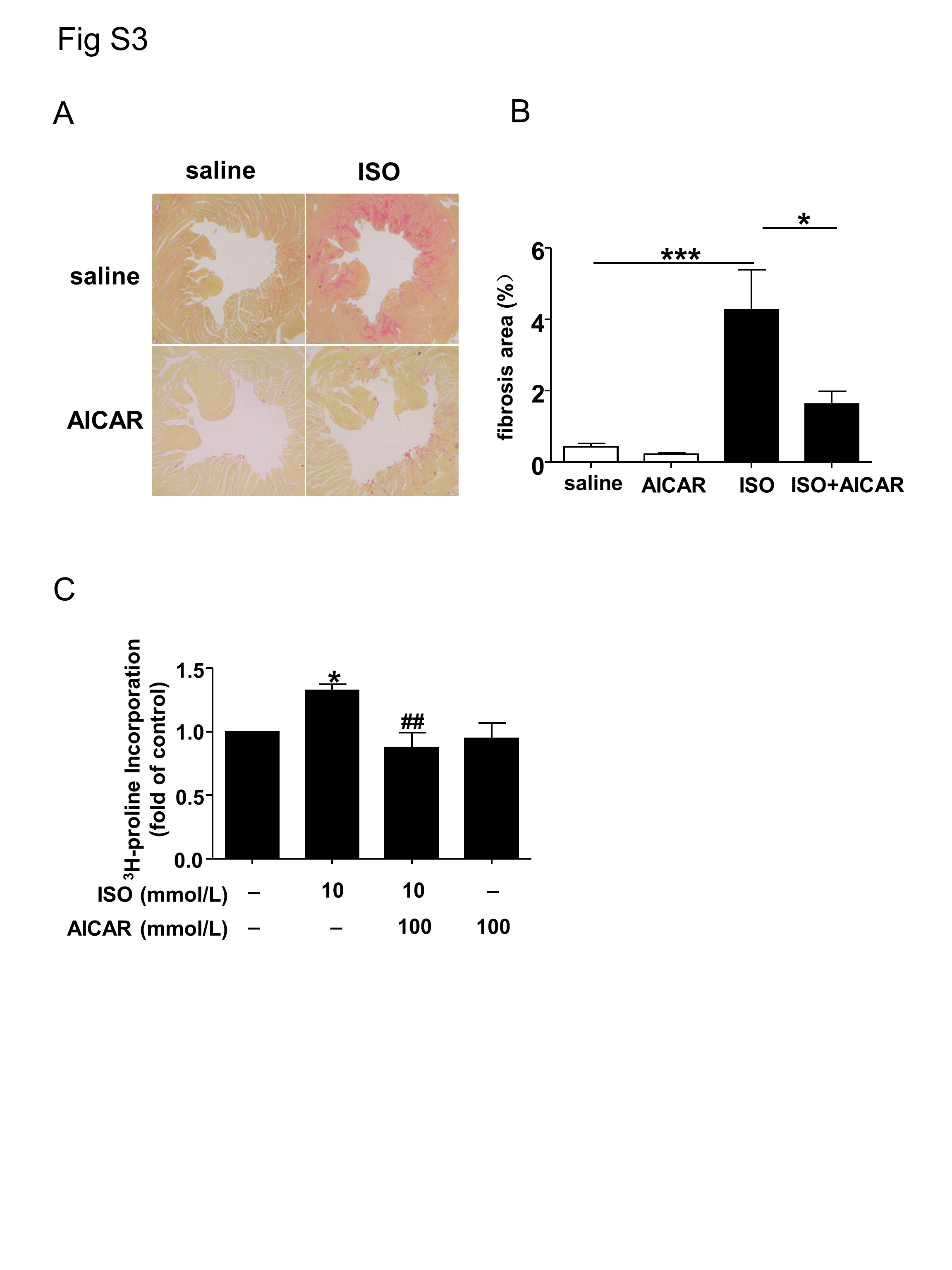

Supplement: S3 Fig — Male AMPKα2+/+ mice (10-week old) were pretreated with AICAR (250mg/kg/d) for 3 days and then treated with ISO (5 mg/kg/day) or vehicle (saline) for 7 days. (A) Representative micrographs of Sirius red-stained sections of the left ventricle. (B) Quantification of mean cardiac interstitial collagen content from Sirius red-stained sections (n = 5–8). *P<0.05, ***P<0.001. Data are mean±SEM. (C) AICAR decreased ISO-induced 3H-proline incorporation in isolated adult mouse cardiac fibroblasts (CFs). CFs were pretreated with AICAR (10−4 mol/L) for 30min and then treated with ISO (10-5mol/L). L-[2,3-3H] proline were then supplied for 48 hours (n = 4). *P < 0.05 vs. con; ## P < 0.01 vs. ISO. Data are mean±SEM. (TIF) [file pone.0129971.s003.tif]

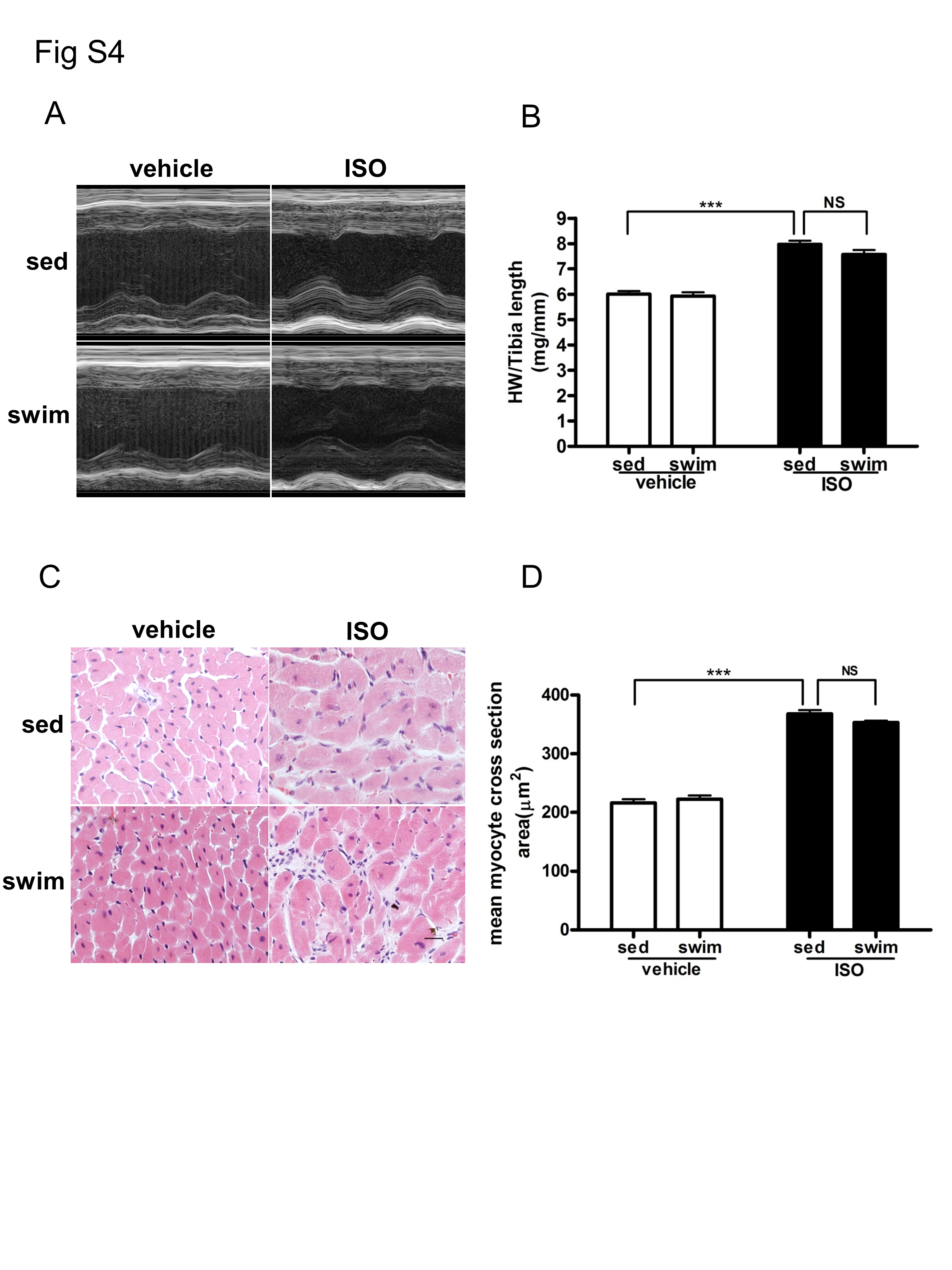

Supplement: S4 Fig — (A) Representative micrographs of echocardiography. (B) The ratio of heart weight (HW) to tibia length (TL) (n = 12). (C) Representative micrographs of HE-stained sections of the left ventricle (LV) (bar = 20 μm). (D) Quantification of mean myocyte cross section area from HE-stained sections (n = 7). *** P<0.001 sed+ISO vs. sed+vehicle; NS, not significant. Data are mean±SEM. (TIF) [file pone.0129971.s004.tif]

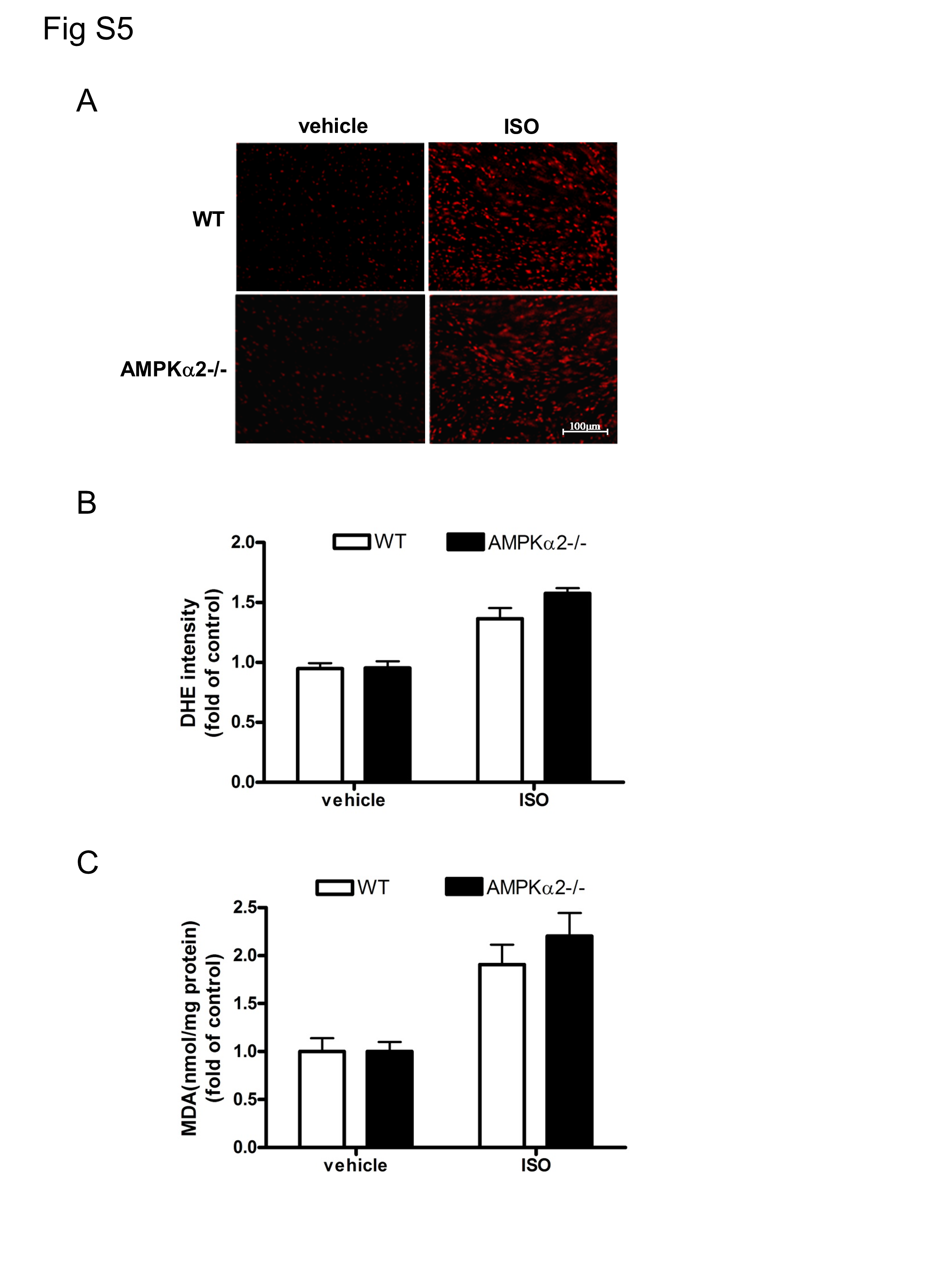

Supplement: S5 Fig — (A) Fluorescent microscopy of representative DHE staining for ROS level in LV sections (bar = 100 μm). (B) Quantification of fluorescence intensity of DHE in LV sections (n = 6). (C) Malonaldehyde (MDA) content in myocardial tissue (n = 6). Data are mean±SEM. (TIF) [file pone.0129971.s005.tif]

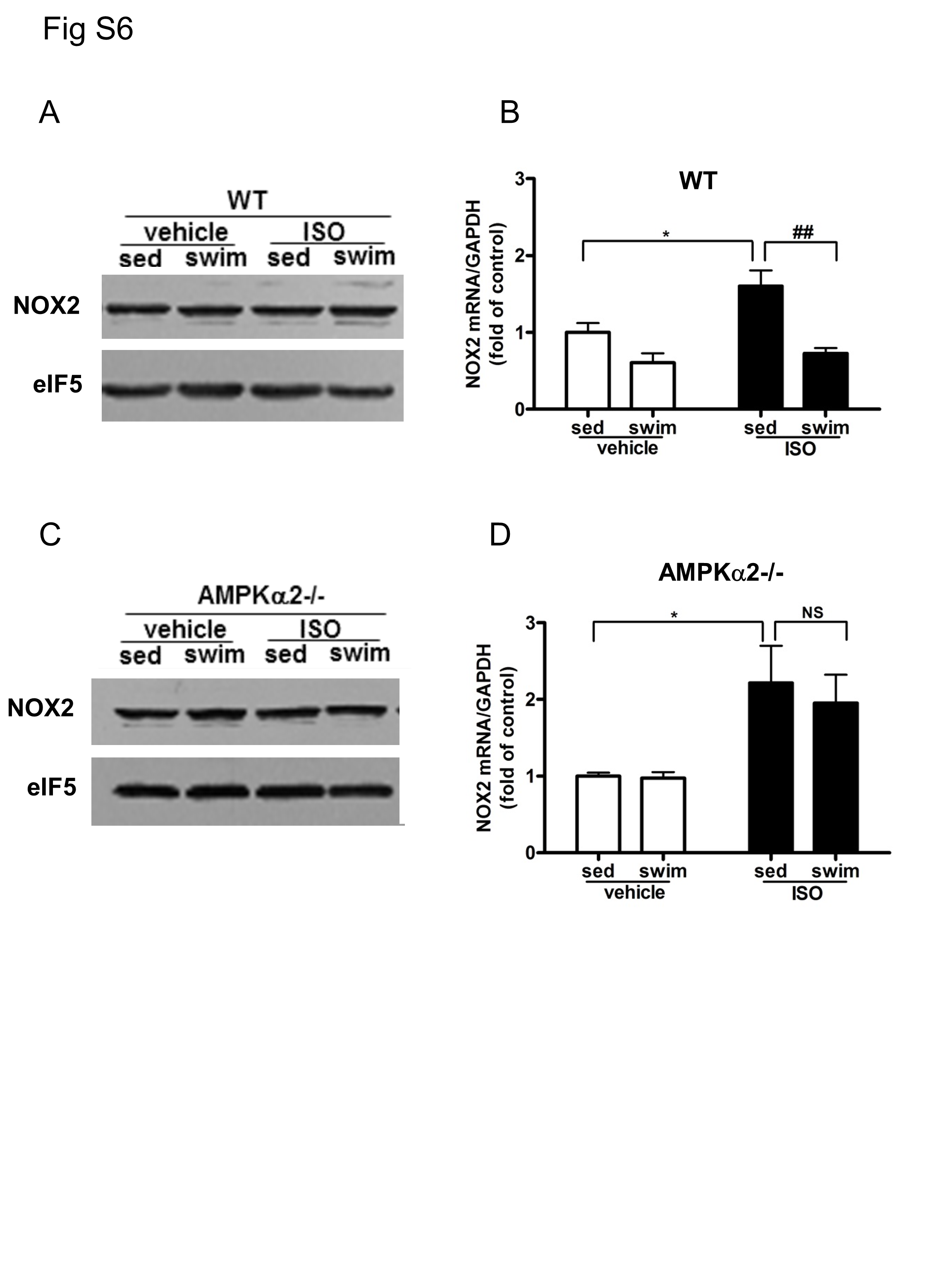

Supplement: S6 Fig — (A) Western blot analysis of myocardial protein levels of NOX2 and eIF5 for AMPKα2+/+ mice. (B) RT-PCR analysis of mRNA expression of NOX2 normalized to that of GAPDH for AMPKα2+/+ mice (n = 5). * P<0.05, sed+ISO vs. sed+vehicle; ## P<0.01, swim+ISO vs. sed+ISO. (C) Western blot analysis of myocardial protein levels of NOX2 and eIF5 for AMPKα2-/- mice. (D) RT-PCR analysis of mRNA expression of NOX2 normalized to that of GAPDH for AMPKα2-/- mice (n = 6). * P<0.05, sed+ISO vs. sed+vehicle; NS, not significant. Data are mean±SEM. (TIF) [file pone.0129971.s006.tif]

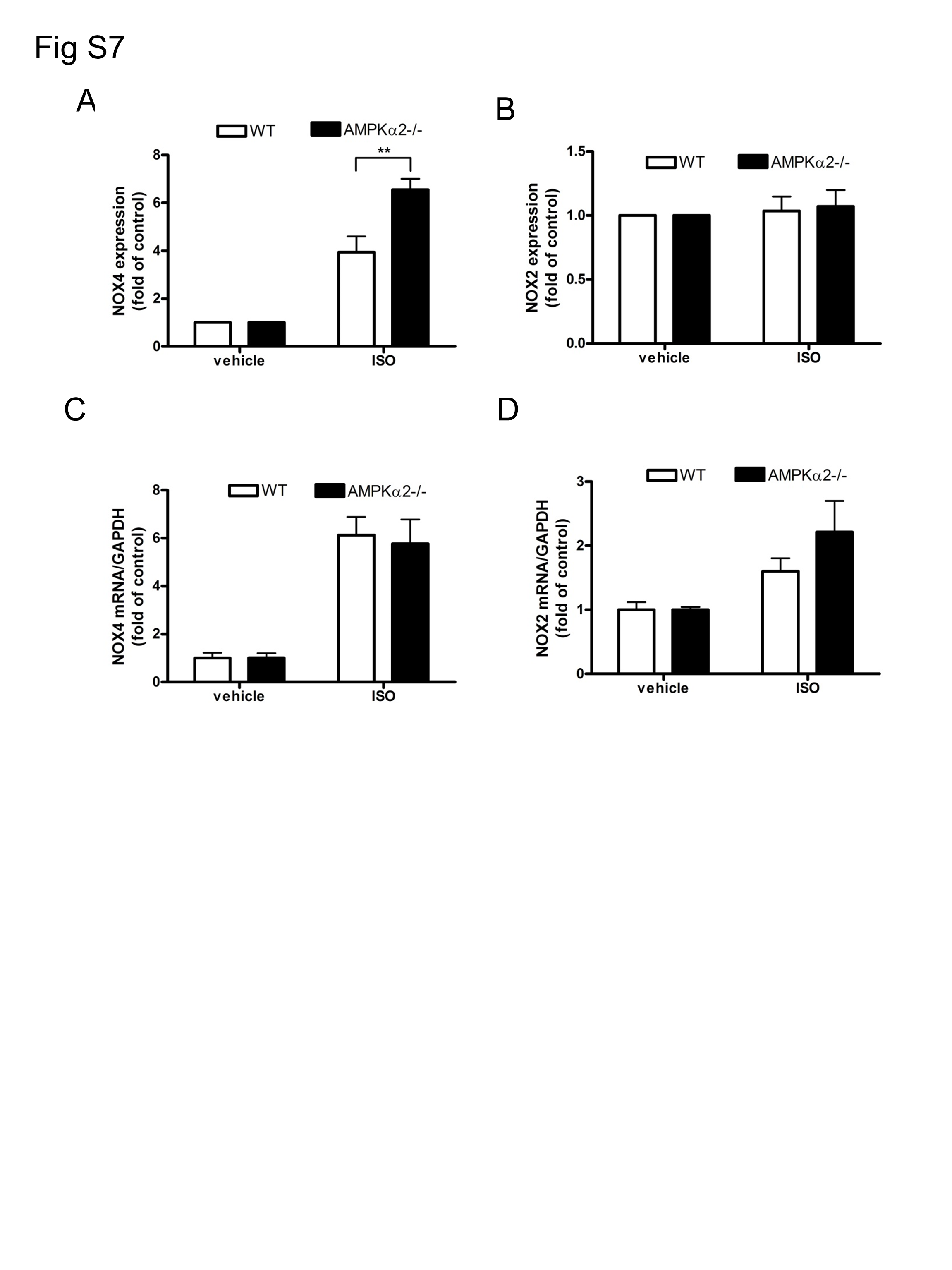

Supplement: S7 Fig — Quantification of Western blot for NOX4 (A) and NOX2 (B) relative to eIF5 (both n = 4). RT-PCR analysis of mRNA expression of NOX4 (C) and NOX2 (D) normalized to that of GAPDH (n = 4~6). ** P < 0.01 ISO-treated AMPKα2-/- vs. AMPKα2+/+ mice. Data are mean±SEM. (TIF) [file pone.0129971.s007.tif]

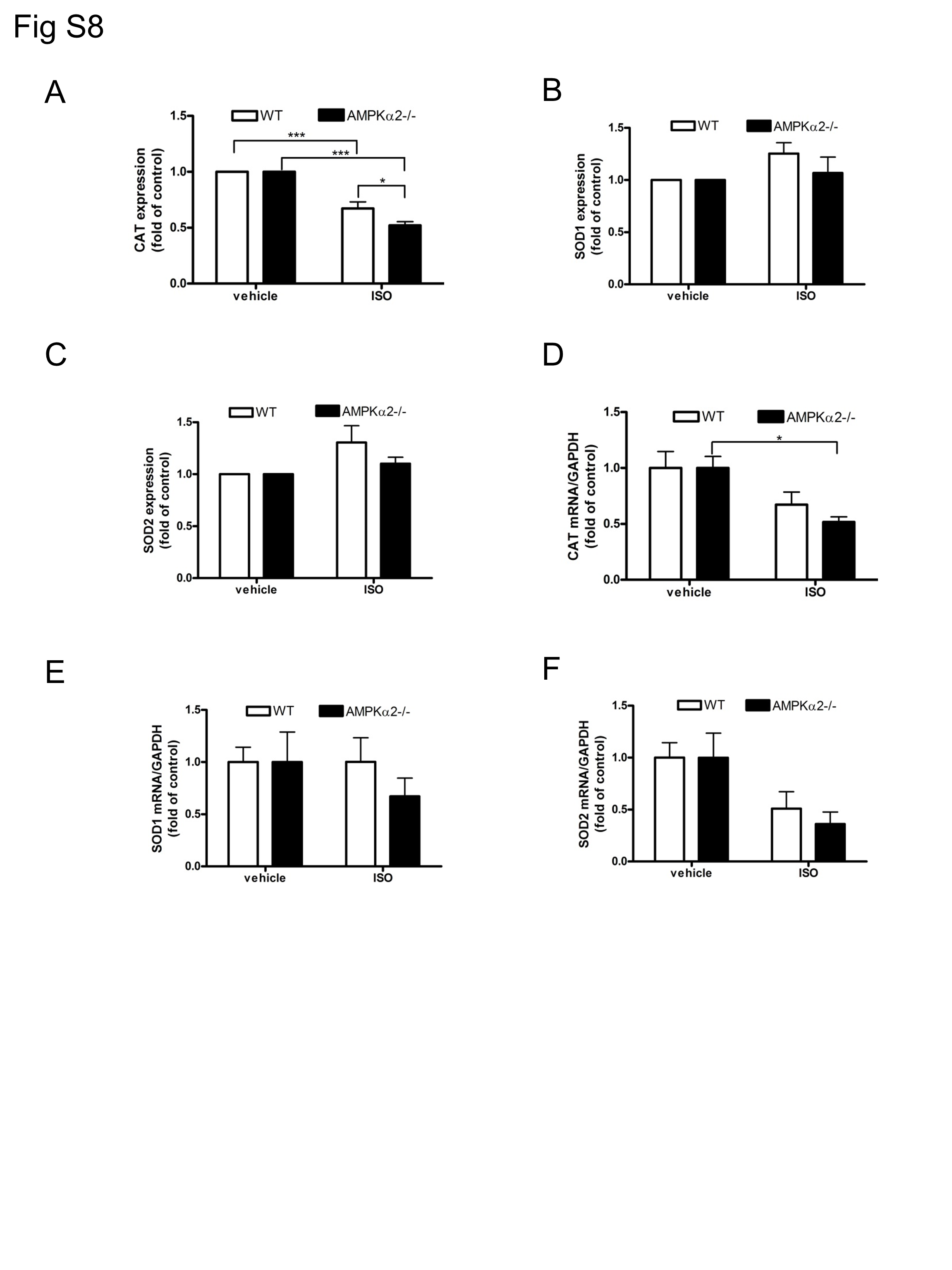

Supplement: S8 Fig — Quantification of Western blot for CAT (A, n = 4), SOD1 (B, n = 5), and SOD2 (C, n = 6) relative to GAPDH. RT-PCR analysis of mRNA expression of CAT (D, n = 5), SOD1 (E, n = 6), and SOD2 (F, n = 5) normalized to that of GAPDH. * P < 0.05, *** P < 0.001. Data are mean±SEM. (TIF) [file pone.0129971.s008.tif]
