# Supplementary material for: Cardiac Fibrosis Alleviated by Exercise Training Is AMPK-Dependent
Source: PLoS One. 2015 Jun 12;10(6):e0129971. doi: 10.1371/journal.pone.0129971 (PMC4466316; doi:10.1371/journal.pone.0129971)
Supplement: S1 Table — (DOC) [file pone.0129971.s009.doc]

**Table S**1. Oligonucleotide primer sequences used for real-time PCR.

| Gene | Forward | Reverse |
| --- | --- | --- |
| Collagen I | 5'-GTAACTTCGTGCCTAGCAACA-3' | 5'-CCTTTGTCAGAATACTGAGCAGC-3' |
| Collagen III | 5'-CCTGGCTCAAATGGCTCAC-3' | 5'-CAGGACTGCCGTTATTCCCG-3' |
| Connective tissue growth factor (CTGF) | 5'-GGGCCTCTTCTGCGATTTC-3' | 5'-ATCCAGGCAAGTGCATTGGTA-3' |
| NADPH oxidase 4 (NOX4) | 5'-ACTTTTCATTGGGCGTCCTC-3' | 5'-AGAACTGGGTCCACAGCAGA-3' |
| NADPH oxidase 2 (NOX2) | 5'-GACCATTGCAAGTGAACACCC-3' | 5'-AAATGAAGTGGACTCCACGCG-3' |
| Superoxide dismutase 1 (SOD1) | 5'-GAGACCTGGGCAATGTGACT-3' | 5'-GTTTACTGCGCAATCCCAAT-3' |
| Superoxide dismutase 2 (SOD2) | 5'- GCGGTCGTGTAAACCTCAAT -3' | 5'- CCAGAGCCTCGTGGTACTTC -3' |
| Catalase (CAT) | 5'-CCAGCGACCAGATGAAGCAG-3' | 5'-CCACTCTCTCAGGAATCCGC-3' |
| GAPDH | 5'-TCCTGGTATGACAATGAATACGGC-3' | 5'-TCTTGCTCAGTGTCCTTGCTGG-3' |
