# Supplementary material for: Cardiac Fibrosis Alleviated by Exercise Training Is AMPK-Dependent
Source: PLoS One. 2015 Jun 12;10(6):e0129971. doi: 10.1371/journal.pone.0129971 (PMC4466316; doi:10.1371/journal.pone.0129971)
Supplement: S1 Methods — (DOCX) [file pone.0129971.s010.docx]

**Supporting Information methods**

**Animal model and drug treatment**

Male AMPKα2^+/+^ mice (10-week old) were pretreated with saline or AICAR (250mg/kg/d) via subcutaneous injection for 3 days, and then were randomized to receive ISO (5mg/kg/d) or vehicle (saline) daily via subcutaneous injection for 7 days.

**Preparation of cardiac fibroblasts**

Adult cardiac fibroblasts (CFs) were isolated from 10-week-old male AMPKα2^+/+^ mice as described. Minced ventricles were digested with 0.01% collagenase II (Worthington, Columbia, NJ, USA). Cells were collected and plated for 2 h at 37℃. Unattached cardiomyocytes were removed. CFs were cultured in Dulbecco’s modified Eagle’s medium (DMEM) with 10% foetal bovine serum (FBS; Hyclone Laboratories, Incorporated, Omaha, NE, USA) at 37℃. Cells in the second passage were used in this experiment. Studies involved CFs grown to 80% confluence and serum starved for 24 h in serum-free medium before treatment.

**Measurement of collagen synthesis**

Collagen synthesis was determined by measuring incorporation of ^3^H-proline as described. In brief, CFs in 24 wells were cultured in serum-free DMEM for 24 h. Cells were then exposed to AICAR (0.1 mmol/L) in the presence or absence of ISO (10 μmol/L). L-[2,3-^3^H] proline (Perkin Elmer, Incorporated Waltham, MA, USA) (0.5 μCi/well) were then supplied for 48 h. Cell extracts were analysed in a liquid scintillation counter. The count represented the amount of newly synthesized type-I collagen.
